# The Relationship Between Spousal Support and Nurse Burnout: A Parallel Dual‐Mediator Model of Work–Family Conflict and Work–Family Facilitation

**DOI:** 10.1155/jonm/7277746

**Published:** 2026-10-21

**Authors:** Juan Li, Sha Lin, Ling Li, Zhen Liang, Qiaofeng Zhang, Xiaoyu Huang, Yan Tan, Yulian Yuan

**Affiliations:** ^1^ The Affiliated Cancer Hospital of Xiangya School of Medicine Central South University/Hunan Cancer Hospital Changsha Hunan China csu.edu.cn; ^2^ Lengshuijiang People’s Hospital Changsha Hunan China; ^3^ The First People’s Hospital of Changsha County Changsha Hunan China

**Keywords:** burnout, human resource management, nursing staff, parallel dual-mediator model, spousal support, work–family conflict, work–family facilitation

## Abstract

**Objective:**

To examine whether work–family conflict (WFC) and work–family facilitation (WFF) act as parallel statistical mediators in the association between spousal support (SS) and burnout among nurses.

**Background:**

Nurse burnout represents a substantial management challenge, contributing to high staff turnover and diminished quality of patient care. SS is a potentially important protective resource for mitigating burnout. Nevertheless, the mechanisms through which SS influences nurse burnout, particularly via positive work–family spillover, remain understudied within nursing populations.

**Methods:**

A cross‐sectional online survey was conducted among 343 married clinical nurses from multiple provinces across China. Validated self‐report questionnaires were used to measure SS, WFC, WFF, and burnout. Path analysis with bootstrapping was performed to test the parallel dual‐mediator model.

**Results:**

The model achieved good fit indices, with *X* ^2^/df = 1.281 and RMSEA = 0.029. SS was negatively associated with WFC (*β* = −0.225, SE = 0.038, 95% CI [−0.300, −0.151]) and positively associated with WFF (*β* = 0.279, SE = 0.022, 95% CI [0.236, 0.321]). Both mediators were significantly associated with burnout. The total indirect effect of SS on burnout was statistically significant (*β* = −0.643, SE = 0.095, 95% CI [–0.835, −0.461]). The indirect pathway operating through WFF accounted for 62.36% of the total indirect effect, which was larger than the 37.64% attributable to the WFC pathway.

**Conclusion:**

These correlational findings suggest that higher SS is associated with lower nurse burnout through both reduced WFC and enhanced positive WFF. The results highlight the critical “resource‐gain” function of family support for married nurses.

**Implications:**

Interventions should not merely target workload reduction but also actively foster SS to promote personal resource development. It is recommended to foster a family‐friendly corporate culture, implement comprehensive initiatives that involve partners, and regard proactive work–family integration as a strategic approach to alleviating nurse burnout.

## 1. Introduction

Burnout, first described by American psychologist Herbert Freudenberger in 1974 [1], refers to a chronic psychological and physical stress state arising from prolonged work‐related pressure, especially within human‐service professions. According to Maslach et al. [2], burnout encompasses three core dimensions: emotional exhaustion, depersonalization, and reduced personal accomplishment. Emotional exhaustion manifests as diminished motivation and enthusiasm for patient care. Depersonalization is characterized by interpersonal detachment and emotional numbness in nursing practice. Reduced personal accomplishment is reflected in feelings of professional inadequacy, persistent self‐doubt, decreased job satisfaction, and impaired work efficiency. A meta‐analysis by Sohrabi et al. reported a 55% pooled prevalence of burnout among nurses. Burnout severely impairs nurses’ physical and mental well‐being and is closely linked to psychological morbidities including anxiety and depression [3]. Furthermore, burnout reduces work engagement and elevates turnover intentions [4, 5]. Consequently, burnout directly compromises clinical care quality and patient safety, threatens healthcare‐system stability, and imposes substantial managerial challenges [6].

Existing research on nurse burnout has predominantly focused on external occupational risk factors such as workload and organizational climate [7, 8], while largely neglecting the family system and spousal support (SS) as a key personal resource. Recent evidence, however, underscores the buffering role of SS against occupational stress. For instance, a physician‐focused study by O’Sullivan et al. demonstrated that greater SS improves work–life integration satisfaction and lowers burnout risk [9]. Similarly, research among married medical residents indicated that intimate‐relationship support serves as a meaningful protective factor [10]. Collectively, these findings suggest that SS—delivered through emotional comfort, household‐responsibility sharing, and recognition of professional worth—plays an important yet insufficiently explored role in mitigating nurse burnout.

Drawing on work–family border (WFB) theory, much prior literature has emphasized the negative consequences of work–family conflict (WFC), positing that incompatibility between work and family roles triggers resource depletion and subsequent burnout [10]. WFC arises from competing work–family demands related to time constraints and interpersonal strain and constitutes a well‐documented burnout risk factor [11, 12]. Previous studies have noted that SS may mitigate burnout by alleviating WFC [5, 13]. According to Conservation of Resources (COR) theory [14], individuals strive to preserve existing resources while pursuing new resources, operating within gain spirals (resources beget additional resources) and loss spirals (resource loss accelerates further resource erosion), highlighting the interconnected nature of personal resources. Beyond conflict‐oriented outcomes, the work–family interface also encompasses positive spillover effects exemplified by work–family facilitation (WFF), whereby family‐derived resources enhance employees’ work‐related performance, vitality, and efficacy [15]. Prior work indicates that adaptive coping strategies can buffer the mental‐health impacts of occupational stress, with SS facilitating such adaptive coping [4, 9]. SS includes emotional, informational, and practical assistance from one’s spouse and represents a vital family resource. Its protective influence extends beyond conflict reduction toward active resource generation, thereby interrupting loss spirals and initiating resource‐gain spirals [16, 17]. Therefore, exclusive focus on the conflict pathway provides an incomplete understanding of SS’s effects. A dual‐path framework incorporating both conflict‐reduction and resource‐gain mechanisms is required to comprehensively understand how family resources shape occupational well‐being. Drawing on both COR theory and WFB theory, we propose an integrated sequential mechanism. From the COR perspective, SS acts as an external family resource that can prevent resource loss and trigger resource‐gain spirals. From the WFB theory perspective, individuals constantly transition across work and family role boundaries. SS may reshape boundary experiences in two divergent ways: it can reduce role incompatibility to mitigate WFC (reducing resource depletion), and it can also generate positive cross‐role spillover to enhance WFF (producing new psychological resources). Changes in WFC and WFF, in turn, determine the degree of resource erosion or resource accumulation, which ultimately shapes nurses’ burnout status. This dual‐path theoretical logic underpins our parallel‐mediation hypothesis.

Accumulating nursing studies have documented that sociodemographic, occupational, and family‐related characteristics may simultaneously shape nurse burnout and work–family interface experiences, and thus represent potential confounders when testing associations between SS and burnout [5–7, 12]. For instance, age, educational attainment, and professional title are known to correlate with nurses’ job resources and burnout severity, whereas frequent night‐shift work and employment type contribute to chronic resource depletion and elevated burnout risk. Subjective income satisfaction, physical health status (e.g., presence of chronic disease), marital duration, and primary childcare‐caregiving responsibilities further influence how married nurses allocate resources across work and family domains, which may distort the true magnitude of relationships among SS, work–family processes, and burnout [8, 9]. Therefore, these variables should be statistically adjusted to reduce confounding bias when exploring the hypothesized mediating pathways.

This study aims to construct a parallel dual‐mediator model to explore whether WFC and WFF statistically mediate the association between SS and burnout among nurses in a cross‐sectional sample. We hypothesize that SS is indirectly associated with burnout via WFC and WFF. Notably, given the cross‐sectional design, this model tests correlational pathways rather than true causal mechanisms. This research provides empirical evidence for the hypothesized associational pathways and enriches understanding of potential protective factors for nurse burnout. It holds significant theoretical value in elucidating the underlying psychological processes. Additionally, it provides a solid foundation for creating targeted interventions for nurses and their spouses, incorporating both “burden‐reduction” and “resource‐gain” strategies.

The following hypotheses were proposed:

 H1: SS is negatively correlated with WFC and burnout and positively correlated with WFF.
 H2: WFC and WFF function as parallel statistical mediators in the association between SS and burnout. Specifically, SS exhibits an indirect statistical association with burnout in parallel via its correlation with WFC (Path 1) and WFF (Path 2).

The theoretical framework of the present study is illustrated in Figure 1.

**FIGURE 1:**
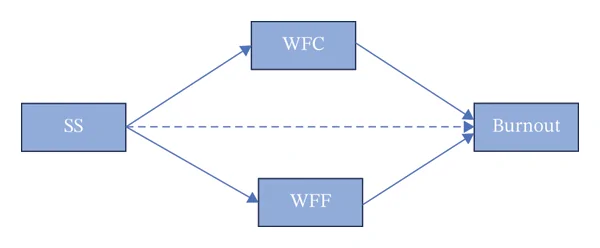
The theoretical framework of this study. Abbreviations: SS = spousal support; WFC = work–family conflict; WFF = work–family facilitation.

## 2. Methods

### 2.1. Design

This cross‐sectional observational study followed the ethical principles outlined in the Declaration of Helsinki for human‐subjects medical research. The study protocol received ethical approval from the Ethics Committee for Clinical Trials of Hunan Cancer Hospital (Approval No. SRSPR‐2025‐253) and adheres to the Strengthening the Reporting of Observational Studies in Epidemiology (STROBE) reporting guideline.

### 2.2. Sample/Setting

Data collection was conducted in October 2025. Participants were recruited using nonprobabilistic convenience snowball sampling via WeChat social networks. Twenty‐five seed nurses were invited to distribute the online questionnaire. This sampling approach carries potential biases, including network homogeneity bias stemming from seed participants’ social circles, self‐selection bias among voluntary online respondents, and under‐representation of nurses with limited WeChat access.

Inclusion criteria: (1) holding a valid nursing practice certificate; (2) minimum of 1 year of clinical nursing experience; (3) currently married; (4) provision of informed consent.

Exclusion criteria: (1) experience of major adverse family events (e.g., bereavement) within the preceding 3 months to avoid acute‐stress interference with support perceptions; (2) inactive clinical duty status due to leave, further training, or other reasons. Additional data‐quality exclusion criteria included incomplete questionnaire responses, patterned responding, and questionnaire completion durations shorter than 360 s.

### 2.3. Data Collection

The opening survey announcement described the study objective of exploring relationships among SS, work–family balance, and burnout and emphasized voluntary participation. After providing electronic informed consent, participants proceeded to the questionnaire. Data collection was implemented via Wenjuanxing (http://www.wjx.cn), a certified Chinese online survey platform complying with national data‐security and privacy regulations. Multiple technical safeguards protected participant anonymity, data integrity, and ethical online‐survey standards:

1. Duplicate‐submission prevention: one response permitted per IP address to avoid repeated submissions from identical devices or networks.
2. Response‐quality filtering: automatic rejection of questionnaires completed in under 360 s based on pilot‐test benchmarks to minimize careless or random responding.
3. Data encryption: SSL/TLS encryption for all data transmission between participant devices and platform servers.
4. Anonymous data handling: no collection of personally identifiable information such as full names or precise residential addresses, except for duplicate‐submission verification; no tracking cookies or third‐party analytics embedded.
5. Access control: password‐protected platform servers; research‐team access restricted through secure login; only aggregated anonymized datasets were downloaded for statistical analysis.
6. Regulatory compliance: platform adherence to China’s Personal Information Protection Law and Cyber Security Law to ensure lawful data management.

### 2.4. Sample Size

For cross‐sectional correlational analyses, a sample size of 10–20 observations per independent variable is recommended. This study contained 22 independent variables; allowing for 20% potential missing‐data inflation yielded a minimum target sample of 275 participants. For structural‐equation‐modeling and bootstrapped mediation analyses, widely accepted guidelines recommend a minimum of 200–300 participants to support stable parameter estimation, bootstrap resampling, and model‐fit evaluation for moderately complex models [18].

### 2.5. Study Measures

#### 2.5.1. Burnout

Burnout was assessed using the Maslach Burnout Inventory (MBI) originally developed by Maslach et al. [2]. The Chinese‐language version translated and validated by Feng et al. [19] represents the primary instrument for occupational burnout assessment in China. The scale comprises 22 items distributed across three subscales, rated on a 7‐point Likert scale ranging from 0 (“*never*”) to 6 (“*every day*”). The emotional‐exhaustion subscale contains nine items; scores ≥ 26 indicate high‐level burnout. The depersonalization subscale includes five items; scores ≥ 9 denote high‐level depersonalization. The eight‐item reduced‐personal‐accomplishment subscale is reverse‐scored, with scores ≥ 14 reflecting high‐level diminished accomplishment. Higher subscale scores correspond to greater burnout severity. The three burnout dimensions were classified by thresholds: depersonalization (≥ 10 points), emotional exhaustion (≥ 27 points), and reduced personal accomplishment (≥ 15 points). Burnout severity categories were defined as mild (one dimension meets threshold), moderate (two dimensions meet criteria), and severe (all three dimensions meet criteria). The original Chinese‐language validation reported Cronbach’s *α* = 0.737; the overall scale Cronbach’s *α* in the present sample was 0.777.

#### 2.5.2. SS

The Work–Family Support Scale developed by Li and Zhao [20] originally includes 30 items across four dimensions: organizational support, leadership support, emotional support, and instrumental support. The family‐domain subscales measuring emotional support and instrumental support were selected to evaluate perceived SS. A total of 10 items were retained for SS assessment: 6 items for emotional support and 4 items for instrumental support. Responses were recorded on a five‐point Likert scale ranging from 1 (“*strongly disagree*”) to 5 (“*strongly agree*”), with higher scores indicating higher perceived SS. These family‐domain subdimensions are theoretically independent components of the original scale and have been adopted in prior nursing‐focused work to measure family‐related support [21, 22]. The 10‐item subset demonstrated Cronbach’s *α* = 0.932 in the current sample, indicating satisfactory internal consistency; the full original scale achieved Cronbach’s *α* = 0.972 in its validation sample.

#### 2.5.3. WFC and WFF

Work–family balance constructs were measured using the scale based on the model proposed by Grzywacz and Marks (2000) [23], yielding separate scores for WFC and WFF. The instrument includes four subscales: WFC (items 1–4), WFF (items 12–14), family‐to‐work conflict (items 8–11), and family‐to‐work facilitation (items 5–7). Participants responded using a five‐point Likert scale ranging from 1 (“*strongly disagree*”) to 5 (“strongly agree”). The Chinese‐language validated version reported Cronbach’s *α* coefficients of 0.870, 0.854, 0.878, and 0.878 for each respective dimension [24]; overall Cronbach’s *α* in this study was 0.864.

### 2.6. Statistical Analysis

All statistical analyses were performed using SPSS 25.0 with a complete‐case analytical approach. All hypothesis tests were two‐tailed with *α* = 0.05. The analytical workflow for evaluating the parallel dual‐mediation hypothesis proceeded sequentially as described below.

#### 2.6.1. Preliminary Analysis

Normality assessments were performed for continuous variables. Skewed variables, such as burnout scores, were reported as Median (interquartile range [IQR]). Demographic data were summarized using frequencies and percentages. The Mann–Whitney U and Kruskal–Wallis H tests were employed to identify demographic and occupational variables, like gender, income satisfaction, and night shifts, that showed significant associations with burnout (*p* < 0 .05). These variables were entered as control variables in subsequent models to minimize confounding effects.

#### 2.6.2. Correlational Analysis

Spearman’s rank‐order correlation coefficients were computed to characterize bivariate relationships among SS, WFC, WFF, and burnout, establishing an empirical foundation for the mediation model.

#### 2.6.3. Testing the Parallel Dual‐Mediation Model

A model‐based analytical strategy was implemented to test the hypothesis that WFC (negative pathway) and WFF (positive pathway) simultaneously mediate the association between SS and burnout. AMOS 24.0 employing maximum‐likelihood estimation was used for model fitting; the PROCESS macro for SPSS (Version 25.0, Model 4) was applied for parallel‐mediation analysis following Preacher and Hayes [25]. Bootstrap resampling with 5000 samples was conducted using the bias‐corrected nonparametric percentile method to generate 95% confidence intervals (95% CI). The independent variable (SS), two mediators (WFC, WFF), outcome variable (burnout), and all prespecified covariates were entered simultaneously. Path coefficients and indirect‐effect estimates were extracted. Mediation‐pathway statistical significance was determined by inspecting bootstrap 95% CIs for indirect effects; total indirect effects represented the sum of the two specific indirect effects and reflected overall mediation magnitude.

## 3. Results

A total of 378 questionnaire submissions were received. 35 responses were excluded for the following reasons: not meeting inclusion criteria (*n* = 7; e.g., nonmarried status, less than 1‐year clinical experience), patterned answering (*n* = 15), and completion time shorter than 360 s (*n* = 13). Participant selection procedures are visualised in Figure 2. After removing invalid responses according to predefined eligibility and quality‐control criteria, 343 valid questionnaires were retained, with an effective response rate of 90.74% (343/378). Participant geographic distribution was as follows: 289 (84.26%) from Hunan Province, 19 (5.54%) from Guizhou Province, 25 (7.29%) from Guangdong Province (Guangzhou), and 10 (2.91%) from other regions. The effective response rate was calculated as the number of valid questionnaires divided by total submitted questionnaires.

**FIGURE 2:**
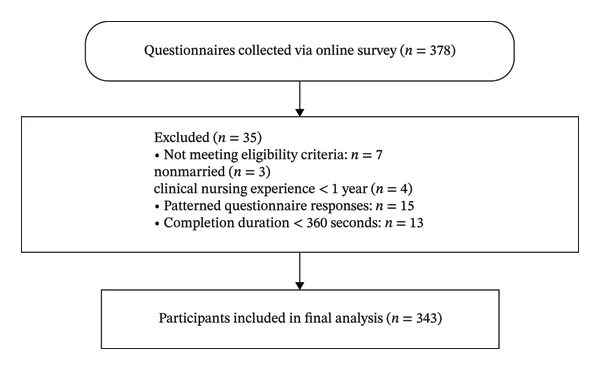
Participant flow diagram.

Given that burnout, perceived SS, WFC, and WFF were all measured via self‐report, common‐method bias represented a potential risk that could inflate observed correlations. Procedural and statistical steps recommended by Liu et al. [26] were applied to mitigate common‐method variance. Procedurally, participants were assured of response anonymity and confidentiality to reduce evaluation apprehension and social‐desirability bias; questionnaire sections separated key constructs to reduce respondents’ awareness of hypothesized variable interconnections. Statistically, Harman’s single‐factor test was performed. The first unrotated factor explained only 13.16% of total variance, well below the 40% threshold proposed by Podsakoff et al. [27]. This finding suggests that no single method factor significantly accounted for the majority of the variance, thus reducing concerns about common method bias.

Burnout score was 47.00 (IQR = 32.00, 59.00). Subscale‐level descriptive statistics were: emotional exhaustion median = 18.00 (IQR = 18.61, 20.50), severe‐level *n* = 64 (18.66%); depersonalization median = 5.00 (IQR = 5.24, 6.22), severe‐level *n* = 78 (22.74%); reduced personal accomplishment median = 21.00 (IQR = 18.57, 20.59), severe‐level *n* = 240 (69.97%). In total, 250 (72.89%) participants exhibited occupational burnout: 148 (43.15%) mild, 71 (20.70%) moderate, and 31 (9.03%) severe burnout.

Table 1 summarizes demographic and occupational characteristics for the 343 participants. The sample was predominantly female (*n* = 328, 95.63%). Most participants were aged 30–39 years (*n* = 212, 61.81%), held a bachelor’s degree (*n* = 287, 83.67%), and occupied senior‐nurse professional titles (*n* = 222, 64.72%). Slightly more than half worked in provincial capital city facilities (*n* = 178, 51.90%), and 263 (76.68%) participants reported more than 10 years of work experience. Regarding work conditions, 167 (48.69%) participants worked 36–40 h weekly; 123 (35.86%) reported approximately four monthly night shifts; 182 (53.06%) expressed neutral income satisfaction. For family‐related characteristics: 152 (44.31%) participants had been married for more than 15 years; 274 (79.88%) cohabited with their spouse; 291 (84.84%) had minor children; 204 (59.48%) reported living with chronic disease; and 198 (57.73%) identified themselves as primary childcare caregivers. Statistically significant differences in burnout distributions were detected across groups defined by age, education level, professional title, employment type, monthly night‐shift count, income satisfaction, chronic‐disease status, marriage duration, and primary‐childcare‐caregiver status (all *p* < 0.05).

**TABLE 1:** Work and life experiences of survey respondents.

Variables	*n* (%)	Burnout, median (*Q*_1_, *Q*_3_)	Statistic	*p*
Total	343 (100)	47.00 (32.00, 59.00)		
Age			*X* ^2^ = 15.80^#^	< 0.001
< 30	41 (11.95)	52.00 (40.00, 62.00)		
30–39	212 (61.81)	48.50 (34.75, 59.25)		
≥ 40	90 (26.24)	39.00 (23.25, 54.00)		
Gender			*Z* = −0.75	0.451
Male	15 (4.37)	49.00 (37.00, 60.00)		
Female	328 (95.63)	47.00 (31.00, 59.00)		
Residence			*X* ^2^ = 5.91^#^	0.052
Town	44 (12.83)	41.00 (26.00, 58.25)		
City	121 (35.28)	51.00 (37.00, 60.00)		
Provincial capital	178 (51.90)	46.00 (30.25, 57.00)		
Education			*X* ^2^ = 8.27^#^	0.016
Associate degree or below	26 (7.58)	44.00 (37.00, 57.75)		
Bachelor’s degree	287 (83.67)	48.00 (33.00, 59.00)		
Master’s degree or above	30 (8.75)	36.00 (26.25, 49.75)		
Professional title			*X* ^2^ = 9.69^#^	0.021
Beginner	20 (5.83)	51.50 (38.50, 60.00)		
Junior	51 (14.87)	49.00 (37.00, 59.50)		
Senior	222 (64.72)	47.00 (31.00, 59.00)		
Deputy Director or above	50 (14.58)	39.00 (22.00, 52.75)		
Hospital level			*X* ^2^ = 2.80^#^	0.247
Level 2 or below	61 (17.78)	41.00 (27.00, 58.00)		
Level 3, Grade B	36 (10.50)	46.00 (31.50, 60.25)		
Level 3, Grade A	246 (71.72)	48.00 (34.25, 59.00)		
Weekly working hours(h)			*X* ^2^ = 3.35^#^	0.341
≤ 35	51 (14.87)	48.00 (26.50, 58.50)		
36–40	167 (48.69)	46.00 (33.50, 57.00)		
41–45	81 (23.62)	48.00 (31.00, 59.00)		
≥ 46	44 (12.83)	49.00 (37.00, 63.50)		
Employment			*X* ^2^ = 9.93^#^	0.019
Formal	167 (48.69)	44.00 (27.50, 57.00)		
Temporary	93 (27.11)	51.00 (38.00, 63.00)		
Labor dispatch	57 (16.62)	41.00 (33.00, 55.00)		
Others	26 (7.58)	52.00 (37.25, 61.00)		
Monthly income (RMB)			*X* ^2^ = 5.85^#^	0.119
< 5000	56 (16.33)	51.00 (36.75, 61.00)		
5000–10000	224 (65.31)	47.00 (32.00, 59.00)		
10,000–20000	60 (17.49)	43.50 (28.75, 56.00)		
> 20,000	3 (0.87)	27.00 (24.00, 38.50)		
Monthly night‐shift count			*X* ^2^ = 18.77^#^	< 0.001
0	87 (25.36)	39.00 (24.50, 54.00)		
1–4	123 (35.86)	44.00 (30.50, 57.00)		
5–8	97 (28.28)	53.00 (36.00, 62.00)		
≥ 9	36 (10.50)	52.50 (39.75, 62.25)		
Income satisfaction			*X* ^2^ = 52.42^#^	< 0.001
Very dissatisfied	15 (4.37)	61.00 (52.00, 66.50)		
Dissatisfied	46 (13.41)	59.00 (47.00, 64.75)		
General	182 (53.06)	48.00 (35.00, 58.75)		
Satisfied	88 (25.66)	38.00 (26.00, 52.50)		
Very satisfied	12 (3.50)	18.00 (10.75, 39.00)		
Chronic‐disease status			*Z* = −3.54	< 0.001
No	139 (40.52)	53.00 (36.50, 62.00)		
Yes	204 (59.48)	42.00 (28.00, 57.00)		
Marriage duration (years)			*X* ^2^ = 15.52^#^	0.001
< 1	17 (4.96)	58.00 (47.00, 68.00)		
1–5	73 (21.28)	54.00 (38.00, 61.00)		
6–10	101 (29.45)	43.00 (31.00, 58.00)		
> 10	152 (44.31)	42.00 (26.00, 56.00)		
Spouse‐cohabitation status			*X* ^2^ = 3.01^#^	0.222
Living together	274 (79.88)	46.50 (31.00, 58.00)		
Separation	57 (16.62)	51.00 (37.00, 61.00)		
Others	12 (3.50)	54.00 (35.75, 62.25)		
Minor‐children status			*Z* = −1.02	0.310
Yes	291 (84.84)	47.00 (29.50, 59.00)		
No	52 (15.16)	48.00 (38.75, 59.00)		
Primary childcare caregiver			*X* ^2^ = 11.59^#^	0.021
Myself	198 (57.73)	47.50 (29.25, 58.00)		
Spouse	53 (15.45)	39.00 (26.00, 55.00)		
Grandparents	35 (10.50)	52.50 (35.50, 62.25)		
Nannies and others	5 (1.45)	60 (58.75, 71.00)		
Working experience (years)			*X* ^2^ = 4.93^#^	0.177
< 5	19 (5.54)	52.00 (39.50, 62.50)		
6–10	61 (17.78)	48.00 (33.00, 59.00)		
11–15	133 (38.76)	47.50 (33.00, 59.00)		
> 15	130 (37.90)	43.50 (27.00, 57.00)		
Department			*X* ^2^ = 5.07^#^	0.167
Emergency Room/ICU/Operating Room	40 (11.66)	47.50 (37.00, 62.25)		
Internal/General Surgery	190 (55.39)	49.00 (34.00, 59.00)		
Oncology	50 (14.58)	47.50 (31.50, 59.00)		
Others	63 (18.37)	40.00 (24.50, 56.50)		

*Note:* Median (M); *Q*_1_ = first quartile; *Q*_3_ = third quartile; *Z* = Mann–Whitney *U* test statistic.

^#^Kruskal–Wallis *H* test statistic.

Table 2 displays the results of the two‐tailed Spearman’s correlation analyses among the burnout subscales (emotional exhaustion, depersonalization, and reduced personal accomplishment), the SS subscales (perceived emotional support and perceived instrumental support), WFC, and WFF. All correlations were statistically significant (*p*<0.01), with coefficients ranging from −0.517 to 0.821. Notably, SS showed a negative correlation with each burnout subscale and with WFC, while it was positively correlated with WFF (*p*<0.01).

**TABLE 2:** Correlations among the continuous variables.

	Burnout	EE	DP	RPA	PES	PIS	SS	WFC	WFF
Burnout	1.000								
EE	0.821^∗∗^	1.000							
DP	0.783^∗∗^	0.658^∗∗^	1.000						
RPA	0.751^∗∗^	0.330^∗∗^	0.436^∗∗^	1.000					
PES	−0.384^∗∗^	−0.282^∗∗^	−0.259^∗∗^	−0.352^∗∗^	1.000				
PIS	−0.420^∗∗^	−0.297^∗∗^	−0.291^∗∗^	−0.405^∗∗^	0.796^∗∗^	1.000			
SS	−0.407^∗∗^	−0.295^∗∗^	−0.276^∗∗^	−0.383^∗∗^	0.968^∗∗^	0.913^∗∗^	1.000		
WFC	0.545^∗∗^	0.549^∗∗^	0.439^∗∗^	0.352^∗∗^	−0.312^∗∗^	−0.362^∗∗^	−0.336^∗∗^	1.000	
WFF	−0.517^∗∗^	−0.377^∗∗^	−0.310^∗∗^	−0.500^∗∗^	0.578^∗∗^	0.554^∗∗^	0.593^∗∗^	−0.312^∗∗^	1.000

*Note:* DP = depersonalization.

Abbreviations: EE, emotional exhaustion; PES, perceive emotional support; PIS, perceived instrumental support; RPA, reduced personal accomplishment; SS, spousal support; WFC, work–family conflict; WFF, work–family facilitation.

^∗∗^ *p* < 0.01.

The hypothesized parallel dual‐mediator model was evaluated using path analysis while adjusting for key demographic and occupational covariates: age, education, professional title, employment type, monthly night‐shift count, income satisfaction, chronic‐disease status, marriage duration, and primary‐childcare‐caregiver status. Model results are displayed in Figure 3. Table 3 shows that SS significantly predicted both mediators. Specifically, SS exhibited a significant negative statistical association with WFC (*β* = −0.225, SE = 0.038, *p* < 0.001) and a significant positive association with WFF (*β* = 0.279, SE = 0.022, *p* < 0.001). Both WFC and WFF showed strong statistical associations with burnout: WFC was positively associated with burnout (*β* = 1.078, SE = 0.151, *p* < 0.001), whereas WFF was negatively associated with burnout (*β* = −1.439, SE = 0.264, *p* < 0.001). After accounting for the two mediators, the direct path from SS to burnout was no longer statistically significant (*β* = −0.164, SE = 0.129, *p* = 0.202).

**FIGURE 3:**
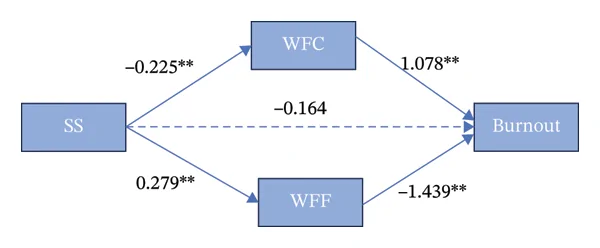
Path analysis results. Note:^∗∗^ *p* < 0.010; abbreviations: SS = spousal support; WFC = work–family conflict; WFF = work–family facilitation.

**TABLE 3:** Results of path coefficients.

Path	*β*	SE	95% CI
SS‐‐WFC	−0.225	0.038	[−0.30∼−0.151 ]
SS‐‐WFF	0.279	0.022	[0.236∼0.321 ]
SS‐‐Burnout	−0.164	0.129	[−0.417, 0.089 ]
WFC‐‐Burnout	1.078	0.151	[0.781, 1.375 ]
WFF‐‐Burnout	−1.439	0.264	[−1.959, −0.919 ]

*Note:* Adjusted for age, education level, professional title, employment status, monthly night‐shift count, income satisfaction, chronic‐disease presence, marriage duration, and primary‐childcare‐caregiver status; *β* = unstandardized regression coefficient; Bootstrap with 5000 resamples for indirect‐effect estimation.

Abbreviations: 95% CI = 95% confidence interval; SS = spousal support; SE = standard error; WFC = work–family conflict; WFF = work–family facilitation.

Mediation‐analysis outputs are presented in Table 4 and demonstrate statistically significant indirect associational pathways. It should be emphasized that these indirect effects are derived from cross‐sectional data and reflect correlational patterns rather than definitive causal pathways; reciprocal or reverse relationships remain plausible. Residual covariance between WFC and WFF was permitted based on work–family interface theoretical reasoning and modification‐index suggestions. This specification yielded a well‐fitting revised model: *X* ^2^/df = 1.281 (< 3), CFI = 0.924, RMSEA = 0.029 (< 0.05), TLI = 0.996, IFI = 0.999, GFI = 0.998, AGFI = 0.981, collectively indicating satisfactory model fit. The total indirect effect of SS on burnout via WFC and WFF combined was *β* = −0.643, SE = 0.095, 95% CI [−0.835, −0.461]. The specific indirect effect transmitted through WFC was *β* = −0.243, accounting for 37.64% of the total indirect effect (95% CI [−0.358, −0.143]). The specific indirect effect via WFF was *β* = −0.401, representing 62.36% of the total indirect effect (95% CI [−0.559, −0.257]). Both indirect pathways were statistically significant because their bias‐corrected bootstrap 95% CIs excluded zero.

**TABLE 4:** Indirect effect analysis results.

Indirect pathway	*β*	SE	95% CI
Via WFC	−0.243	0.055	[−0.358, −0.143]
Via WFF	−0.401	0.077	[−0.559, −0.257]
Total	−0.643	0.095	[−0.835, −0.461]

*Note:* Adjusted for age, education level, professional title, employment status, monthly night‐shift count, income satisfaction, chronic‐disease presence, marriage duration, and primary‐childcare‐caregiver status. *β* = unstandardized regression coefficient; Bootstrap with 5000 resamples for indirect‐effect estimation.

Abbreviations: 95% CI = 95% confidence interval; SE = standard error; WFC = work–family conflict; WFF = work–family facilitation.

## 4. Discussion

### 4.1. Key Findings and Interpretation

This study constructed a parallel dual‐mediator model to explore the mediating roles of WFC and WFF in the association between SS and nurse burnout. Overall, our findings indicate that SS exerts a protective effect on nurse burnout through dual work–family pathways, which further enriches the WFB theory in Chinese nursing populations.

Consistent with prior published work [28, 29], this study observed a 72.9% prevalence of burnout among participating nurses, reinforcing that healthcare professionals face high occupational‐stress‐related risk. Among the three burnout dimensions, reduced personal accomplishment was most prevalent, affecting 70% of respondents. This observation aligns with the umbrella‐review conclusions reported by Getie et al. [30], who identified diminished personal accomplishment as a frequent burnout feature among nurses. Within the Chinese context, this pattern may partly stem from stereotyped perceptions of nursing as auxiliary clinical work, career‐progression stagnation, and difficult clinician‐nurse interpersonal dynamics [31]. Demographic‐stratified analyses demonstrated that nurses holding higher professional titles, advanced educational attainment, and longer professional tenure tended to exhibit lower burnout levels [32, 33]. This observation is consistent with COR theory expectations, as these characteristics correspond to greater job autonomy, enhanced resource access, and improved coping capacity [34]. Conversely, frequent night‐shift work and low income satisfaction correlated with elevated burnout, reflecting sustained resource depletion, replicating previous research results [35]. One noteworthy observation was that nurses whose spouses assumed primary childcare responsibilities presented significantly lower burnout levels. O’Sullivan et al. similarly documented links between household‐childcare‐duty allocation and burnout risk [9], highlighting the importance of instrumental SS. These results emphasize the necessity of accounting for such household‐role variables in future analyses to mitigate confounding.

Correlational analyses showed that SS was significantly negatively correlated with WFC and burnout [36], and significantly positively correlated with WFF [16], which supported Hypothesis H1. These findings suggest that SS may operate both as a “burden‐reducer” and a “resource‐enabler.” Further examination of burnout dimensions demonstrated that emotional exhaustion had the strongest correlation with WFC (*r* = 0.549). This pattern implies that resource depletion stemming from heavy emotional labor may be linked to negative work‐to‐family spillover, which interferes with family‐role performance and coincides with elevated WFC [37]. By contrast, reduced personal accomplishment yielded the strongest negative correlation with WFF (*r* = −0.500), pointing to the compensatory potential of the family domain. Positive experiences and perceived recognition within family settings may buffer against low self‐worth associated with professional marginalization [10]. Additional analysis of support subdimensions revealed that instrumental support had a slightly stronger negative correlation with WFC (*r* = −0.362) relative to emotional support (*r* = −0.312). On the other hand, emotional support exhibited a marginally stronger positive correlation with WFF (*r* = 0.578) compared with instrumental support (*r* = 0.554). Such subtle discrepancies hint at divergent underlying mechanisms: instrumental support may provide “defensive” assistance by mitigating objective resource shortages (e.g., time and household labour) that co‐occur with conflict, whereas emotional support, via empathic listening and affirmation, may deliver “enabling” assistance by nurturing positive psychological resources and self‐worth. These observed correlational patterns lay an empirical basis for testing the proposed parallel dual‐mediator model with WFC and WFF as mediators.

Path and mediation analyses robustly supported Hypothesis H2, elucidating how SS alleviates nurse burnout. Both WFC and WFF acted as mediators in the relationship between SS and burnout [9, 38], with both pathways demonstrating full mediation. Consistent with prior international studies, higher SS effectively buffers occupational burnout by reducing WFC [9, 13]. Family emotional and instrumental resources help nurses relieve role pressure, conserve personal psychological resources, and reduce resource depletion caused by WFC [14, 26]. Furthermore, our study confirmed the positive spillover effect of SS on WFF, which is consistent with previous resource‐based work–family research [15, 23]. Compared with Western studies that mainly emphasize the conflict pathway [13], the present study further highlights the unique facilitating pathway in Chinese cultural contexts. Traditional social customs usually assign most household chores and dependent care responsibilities to women, which strengthens the protective function of SS in the work–family interface [20]. In collectivist family cultures, spousal participation and family support can significantly enhance nurses’ positive work experiences, improve work–family balance, and ultimately alleviate burnout [12, 24].

Notably, the dual mediating results should be interpreted cautiously. Although the parallel mediation model demonstrated acceptable model fit and significant indirect pathways, all data were collected through a cross‐sectional design. Therefore, the observed associations cannot confirm strict causal relationships. The significant mediating effects only reflect statistical correlational patterns, and reciprocal or reverse causality is plausible [39]. For example, lower burnout may also enable nurses to better perceive SS and generate higher WFF. In addition, the present study found that the WFF pathway contributed a larger proportion of the total indirect effect (62.36%) than the conflict pathway (37.64%). This finding differs from some Western studies that reported conflict dominance [13], indicating that family positive resource compensation may be more prominent among married Chinese nurses. This cultural discrepancy suggests that burnout intervention for Chinese nurses should pay more attention to positive family resources rather than only focusing on reducing WFC.

### 4.2. Implications for Nursing Management

Healthcare workers commonly experience elevated WFC, insufficient emotional support, and limited coping strategies for managing emotional‐exhaustion symptoms. Interventions limited to either individual‐level or organizational‐level measures alone may be insufficient to mitigate burnout risks [10, 29]. Accordingly, several practical recommendations for nursing‐management practice emerge from these findings.

First, partner‐inclusive support programming should be prioritized. Healthcare institutions may organize regular online–offline workshops for nurses’ spouses. These sessions should move beyond mere household‐duty‐division discussion and focus on training spouses to deliver high‐quality emotional validation and constructive feedback, thereby strengthening the WFF‐oriented resource‐gain pathway.

Second, empowerment‐focused intervention approaches deserve promotion. Program initiatives such as “Skill Transfer and Value Discovery” not only enhance nurses’ and their family members’ ability to leverage resources but also focus on developing nurses’ perceptual skills. For nurses in different departments and career stages, targeted intervention strategies can also be designed to ensure that empowerment interventions match the actual work–family balance needs of different groups, effectively helping nurses alleviate work burnout. Additionally, encouraging “family‐appreciation rituals” can translate implicit family support into explicit WFF‐related positive experiences.

Third, tiered risk‐targeted management is needed for high‐risk subgroups. Nurses experiencing heavy night‐shift burdens, low income satisfaction, or major primary‐caregiving responsibilities require practical interventions. Flexible shift‐scheduling policies, on‐site childcare‐support resources, and financial‐wellness programming can reduce resource‐depletion‐related strain [17]. Furthermore, “spouse‐involved career coaching” for junior‐career nurses may interrupt adverse resource‐loss spirals. Tailored support strategies are recommended: for nurses primarily suffering from emotional exhaustion, strengthening instrumental SS to alleviate WFC is key; for nurses with prominent reduced‐personal‐accomplishment symptoms, enhancing emotional SS to foster WFF represents the priority direction.

### 4.3. Strengths and Limitations

Strengths: This study has several notable strengths. First, it focuses on nurse burnout, a pressing clinical‐management challenge closely linked to staff retention, job satisfaction, and patient care quality. Second, the present study proposes a novel parallel dual‐mediator model incorporating both WFC and WFF within COR theory. This dual‐path framework provides a valuable perspective for understanding how family‐derived resources shape occupational well‐being among married nurses, moving beyond research that predominantly emphasizes only conflict‐related mechanisms. Third, robust analytical strategies including bootstrapped mediation via the PROCESS macro and comprehensive model‐fit evaluation were implemented to test hypothesized indirect pathways. Finally, the study translates statistical findings into actionable practical recommendations for nursing management, highlighting the value of partner‐inclusive support interventions and family‐friendly organizational policy design.

This study has several notable limitations that should be acknowledged, alongside corresponding directions for future research. First, the cross‐sectional study design precludes rigorous causal inference. Although the parallel dual‐mediator model yielded significant indirect effects, all observed associations are statistical correlations rather than definitive causal pathways, and the temporal ordering of key variables cannot be confirmed. Reciprocal or reverse relationships are theoretically plausible; for instance, severe job burnout may conversely reduce SS and exacerbate negative work–family experiences. To address this limitation, future studies could adopt longitudinal tracking, experience‐sampling, or daily‐diary designs to verify the causal mechanisms and temporal sequences of the hypothesized model. Second, all study data were collected through single‐source self‐report questionnaires. Although we implemented procedural controls (anonymity, questionnaire segmentation) and performed Harman’s single‐factor test to evaluate common‐method bias, residual common‐method variance cannot be completely eliminated. Subjective self‐perception may introduce inherent reporting bias, restricting the objectivity of the research findings. Future research can optimize data collection by adopting a multi‐source evaluation framework, including partner‐reported SS and objective institutional indicators such as nurse turnover rate, to enhance result credibility. Furthermore, SS, WFF, and emotional‐resource‐related constructs have partial conceptual overlap; semantic similarities between measurement items may inflate correlation magnitudes, which should be considered when interpreting effect sizes. Third, this study adopted convenience snowball sampling via WeChat platforms, which may lead to self‐selection bias and network homogeneity bias. The recruited participants were limited to the social network of seed respondents, so the sample cannot fully represent all married clinical nurses across the country. The nonprobabilistic sampling method limits the nationwide generalizability of the findings, and probabilistic sampling strategies are recommended for future large‐scale, representative investigations. Fourth, the sample consists exclusively of Chinese married nurses with a female predominance, bringing obvious cultural and demographic constraints on the generalizability of the research conclusions. This unique cultural context explains the dominant contribution of the WFC pathway in this study, which differs from findings based on Western populations. Nevertheless, this study failed to quantitatively measure cultural values, gender‐role attitudes, and subjective caregiving pressure, which may moderate the predictive effect of SS on work–family states and burnout. Future cross‐cultural and multigroup comparative studies are required to systematically explore how cultural differences and gender norms shape individuals’ work–family resource allocation behaviors, and further verify the cross‐cultural invariance of the dual‐mediator model.

## 5. Conclusion

In conclusion, this cross‐sectional study identifies statistical evidence for dual associational pathways linking SS to nurse burnout via WFC and WFF. SS is correlated with lower burnout in association with both reduced resource‐depleting WFC and enhanced resource‐generating WFF. The pronounced impact of the promotion pathway underscores a shift from merely alleviating burdens to actively fostering benefits. Effectively addressing job burnout among nursing staff necessitates a comprehensive strategy that includes bolstering SS, minimizing WFC, and encouraging WFF. By implementing these strategies, human resource management can cultivate a supportive and balanced work environment that ultimately benefits both employees and the healthcare system.

## Author Contributions

Yan Tan and Juan Li contributed to the conception and design of the study. Zhen Liang, Yulian Yuan, Qiaofeng Zhang, and Xiaoyu Huang collected the data. Ling Li and Sha Lin entered and checked the data. Yulian Yuan and Juan Li analyzed and interpreted the data. Juan Li was the major contributor in writing the manuscript. Yulian Yuan and Yan Tan critically revised the manuscript for intellectual content.

## Funding

This study was supported by Hunan Provincial Natural Science Foundation of China (2026JJ81863).

## Disclosure

All authors read and approved the final manuscript.

## Ethics Statement

This study was performed in line with the principles of the Declaration of Helsinki. Approval was granted by the Ethics Committee of Hunan Cancer Hospital (Approval No. SRSPR‐2025‐253).

## Consent

Informed consent was obtained from all individual participants included in the study.

## Conflicts of Interest

The authors declare no conflicts of interest.

## Data Availability

The data that support the findings of this study are available from the corresponding author upon reasonable request.
